# Supplementary material for: An improved efficient anonymous authentication with conditional privacy-preserving scheme for VANETs
Source: PLoS One. 2021 Sep 10;16(9):e0257044. doi: 10.1371/journal.pone.0257044 (PMC8432652; doi:10.1371/journal.pone.0257044)
Supplement: Attachment — Submitted filename: LIST_OF_REVISIONS_PONE-D-21-19203-Hwang.pdf [file pone.0257044.s001.pdf]

LIST OF REVISIONS

Reference No.: PONE-D-21-19203

Title: An improved efficient anonymous authentication with conditional privacy-preserving scheme for VANETs

Author(s): Eko Fajar Cahyadi, Min-Shiang Hwang

The referees' comments concerning our manuscript were extremely helpful to us in preparing a clearer version. We have revised our manuscript according to the referees' and editor's suggestions. We have marked in blue the parts that have been changed in the revised manuscript or explain. The main revised parts are briefly described as follows:

****

Reviewer 1: Comments to the Author

1. Several grammar mistakes of English exist in this paper, which need to be carefully checked and revised.

Authors' reply:

Thanks for the referee's comment. We have checked and revised some grammatical errors in our manuscript. In addition, we have tried our best to proofread it.

---

2. The format of your paper is wrong. Please check your format for this journal using their template.

Authors' reply:

Thanks for the referee's comment. We use the template from the PLOS ONE journal's website for our manuscript. https://journals.plos.org/plosone/s/latex.

---

3. Please cite the following related important papers in this area.

   a) DOI: 10.1109/IVS.2015.7225898

   b) https://doi.org/10.1007/s11276-013-0543-7

Authors' reply:

Thanks for the referee's comment. Yes, we have included both "https://doi.org/10.1109/IVS.2015.7225898" and "https://doi.org/10.1007/s11276-013-0543-7" on the Introduction section and References [23] and [25]. We categorized the first article as a symmetric cryptography-based authentication scheme. Meanwhile, the other one is included as the ID-based signature scheme.

Reviewer 2: Comments to the Author

1. I think, there is a room to improve the English of this paper. I suggest authors to completely proof-read this paper to improve its readability.

Authors' reply:

Thanks for the referee's comment. We have checked and revised some grammatical errors in our manuscript. In addition, we have tried our best to proofread it.

---

2. I found there are two relevant schemes in VANETs authentication which have been overlooked by authors and should be discussed in the introduction and if possible should be compared with:

https://ieeexplore.ieee.org/abstract/document/6576161
https://ieeexplore.ieee.org/abstract/document/7047920

These two papers are seminal in ID-based verification of VANETs and could be discussed in the introduction section also to give a clear background of the research work.

Authors' reply:

Thanks for the referee's comment. Both papers you suggested above are important, and we also added them in References [26] and [28]. We have included both suggested articles in the Introduction section and compare them in the Security Analysis and Performance Analysis sections. In the Introduction section, both articles included an ID-based signature scheme.

---

3. The digital authentication field has revolutionized during the last 2 decades and there are so many new innovations by using cloud and biometrics authentication. I suggest authors to **discuss how privacy, cloud and biometrics have impacted the authentication field and what benefits it has brought to the automotive industry** for VANETs. There are some following papers which could give a good background of it and can be discussed in the revision:
   https://www.sciencedirect.com/science/article/abs/pii/S1084804512001890
   https://www.sciencedirect.com/science/article/abs/pii/S1084804518301309
   https://www.tandfonline.com/doi/abs/10.4103/0256-4602.50703

Authors' reply:

Thanks for the referee's comment. Yes, we have discussed the above-suggested papers in the second and third paragraphs (Page 2) of the Introduction section. We have added references in [11], [13], and [15].

---

4. Its better to improve and revise the conclusion section of this paper.

Authors' reply:

Thanks for the referee's comment. We have rewritten the Conclusion section.

Previous version:

"This article has shown that Kazemi *et al.*'s scheme does not provide unlinkability and suffers from a forgery attack that leads to an impersonation attack. The adversary can obtain the secret parameter AK_{u_i} through Euclid's algorithm by linking two or more messages msg sent by the same user u_i. To overcome these drawbacks, we have proposed our improvements to security vulnerabilities by giving the scheme an immunity to unlinkability impendency, leading to forgery and impersonation attacks."

Revised version:

"In this paper, we proposed an improvement towards Kazemi *et al.*'s authentication scheme. Our investigation exhibited that an adversary A can derive the user's secret parameter AK_{u_i} through Euclid's algorithm property. Thus, it does not provide traceability and unlinkability, which leads to impersonation attacks. Since introducing two types of improvements, whether encrypts the message M with a shared secret key sk between sender and receiver or put a $Nonce$ in the final message msg, we have proposed improving this scheme and making it secure."

Acknowledgement

The authors would like to thank the anonymous referee for their valuable discussions and comments.
